# Editorial: Mitophagy in health and disease, volume II

**DOI:** 10.3389/fcell.2023.1242664

**Published:** 2023-06-29

**Authors:** Evandro Fei Fang, Nektarios Tavernarakis, Konstantinos Palikaras

**Affiliations:** ^1^ Department of Clinical Molecular Biology, University of Oslo and Akershus University Hospital, Lørenskog, Norway; ^2^ The Norwegian Centre on Healthy Ageing (NO-Age), Oslo, Norway; ^3^ Institute of Molecular Biology and Biotechnology, Foundation for Research and Technology-Hellas, Heraklion, Greece; ^4^ Department of Basic Sciences, School of Medicine, University of Crete, Heraklion, Greece; ^5^ Department of Physiology, Medical School, National and Kapodistrian University of Athens, Athens, Greece

**Keywords:** aging, autophagy, cardiomyopathy, cell death, diabetes, homeostasis, kidney, mitophagy

Mitochondrial selective autophagy, known as mitophagy, is the primary cellular process through which cells regulate the quantity and the quality of mitochondria in response to metabolic and physiological changes. Mitophagy has been the subject of extensive research in the last two decades, uncovering the intricate signaling pathways and the complex mechanisms involved in degradation of superfluous and/or damaged mitochondria. Several mitophagy-related proteins have been identified, revealing a sophisticated regulatory network that preserves mitochondrial function upon developmental, hormonal, and environmental cues. Furthermore, the interconnected communication between these signaling pathways modulate energy metabolism promoting tissue and organ homeostasis. When mitophagy malfunctions, defective mitochondria are accumulated resulting in bioenergetic imbalance, elevated generation of reactive oxygen species (ROS), and chronic inflammation, ultimately leading to cellular death and tissue degeneration. Consequently, mitophagy significantly contributes to cellular physiology, tissue integrity, as well as overall organismal development, healthspan, and survival.

The Research Topic on *Mitophagy in health and disease, Volume II* in *Frontiers in Cell and Developmental Biology* includes a series of 4 articles that shed light on the diverse facets of mitophagy research. The following articles discuss recent advances in the field of mitophagy and mitochondrial metabolism research and provide novel insights into the regulatory mechanisms in various physiological and pathological conditions.

Despite its relatively small size, mitochondrial DNA (mtDNA) is crucial for normal organismal development, function, and survival. Loss of mtDNA integrity has been implicated in the normal aging process, inflammation, and the etiology and pathogenesis of several diseases, including mitochondrial diseases and premature ageing. In their review, Akbari et al. provide an overview of the molecular mechanisms that orchestrate mtDNA repair and transcription underscoring the dynamic functional interactions between the components of these processes. The authors suggest that mtDNA-targeted modulation can be utilized for the development of novel therapeutic interventions to mitigate the negative effects associated with mtDNA instability.

Diabetic cardiomyopathy (DCM) is a significant cause of mortality in individuals with diabetes mellitus (DM) and is characterized by impaired heart function. While mitophagy is crucial for the elimination of damaged mitochondria and preserving cellular homeostasis, in their review, Zheng et al. point out that excessive mitophagy might be detrimental for the myocardium in DCM. The authors suggest that the delicate balance between mitochondrial biogenesis and mitophagy is pivotal for the maintenance of cellular metabolism and cardiac function in individuals with diabetes. In addition, they highlight the need for a better understanding of the precise molecular mechanisms involved in mitophagy and the development of effective interventions to coordinate mitochondrial biogenesis and degradation, thereby preserving energy metabolism and ameliorating cardiac dysfunction in diabetic patients.

Diabetic kidney disease (DKD) is the leading cause of end-stage kidney disease globally and a major microvascular complication associated with diabetes mellitus (DM). In their review, Zhang et al. emphasize the significance of mitophagy in mitigating the risk of DKD, especially in the context of additional pathological conditions, such as COVID-19 infection, which has been associated with an increased incidence of diabetic complications. Moreover, they underscore the crucial role of mitophagy in the maintenance of homeostasis in the glomeruli and tubules. They provide compelling evidence supporting the notion that mitophagy promotes energy metabolism and overall cellular function in the kidneys. The authors suggest that kidney function can be restored in individuals with DKD by enhancing mitochondrial degradation. Overall, the authors highlight the importance of mitophagy in kidney physiology and suggest its stimulation as a potential therapeutic strategy against DKD.

In their research paper, Schiavi et al. investigate the effects of oxygen and iron depletion on cellular and organismal physiology in both the nematode *Caenorhabditis elegans* and mammalian cells. Mild hypoxia and non-toxic levels of iron chelation promote cellular metabolism and enhance organismal survival by inducing mitophagy. The authors investigate the mitophagic capacity of cobalt chloride, an iron competing agent, and discover its neuroprotective effect at sub-lethal doses against hypoxia- or iron depletion-induced cell death in mammalian cells. Moreover, cobalt chloride supplementation extends lifespan and sustains locomotion in several nematode models of neurodegenerative diseases. Taken together, the authors suggest the beneficial effects of cobalt chloride across species via the activation of a hormetic response.

In closing this Editorial, we would like to thank the authors and referees for their invaluable input in assembling this up-to-date Research Topic on the role of mitophagy in health and disease. We anticipate that the assortment of articles encompassed within this Research Topic will serve as a valuable resource and inspire additional research endeavors seeking to unravel the intricate role of mitophagy in the maintenance of cellular, tissue and organismal physiology.

